# Molecular Analysis of Streptomycin Resistance Genes in Clinical Strains of *Mycobacterium tuberculosis* and Biocomputational Analysis of the *Mt*GidB L101F Variant

**DOI:** 10.3390/antibiotics10070807

**Published:** 2021-07-02

**Authors:** Álvaro Rodríguez-García, Rosa E. Mares-Alejandre, Patricia L. A. Muñoz-Muñoz, Samuel Ruvalcaba-Ruiz, Ricardo A. González-Sánchez, Johanna Bernáldez-Sarabia, Samuel G. Meléndez-López, Alexei F. Licea-Navarro, Marco A. Ramos-Ibarra

**Affiliations:** 1 Biotechnology and Biosciences Research Group, Faculty of Chemical Sciences and Engineering, Autonomous University of Baja California, Tijuana 22390, Mexico; alvaro.rodriguez@uabc.edu.mx (Á.R.-G.); lilian.munoz.munoz@uabc.edu.mx (P.L.A.M.-M.); samuelmelendez@uabc.edu.mx (S.G.M.-L.); 2 Clinical Diagnostic Laboratory, General Hospital of Tijuana, Tijuana 22010, Mexico; samuelruvalcaba6@hotmail.com; 3 Department of Biomedical Innovation, Center for Scientific Research and Higher Education at Ensenada, Ensenada 22860, Mexico; ragonzal@cicese.mx (R.A.G.-S.); jbernald@cicese.mx (J.B.-S.); alicea@cicese.mx (A.F.L.-N.)

**Keywords:** *Mycobacterium tuberculosis*, streptomycin resistance, molecular analysis, biocomputational analysis, clinical isolates

## Abstract

Globally, tuberculosis (TB) remains a prevalent threat to public health. In 2019, TB affected 10 million people and caused 1.4 million deaths. The major challenge for controlling this infectious disease is the emergence and spread of drug-resistant *Mycobacterium tuberculosis*, the causative agent of TB. The antibiotic streptomycin is not a current first-line anti-TB drug. However, WHO recommends its use in patients infected with a streptomycin-sensitive strain. Several mutations in the *M. tuberculosis* *rpsL*, *rrs* and *gidB* genes have proved association with streptomycin resistance. In this study, we performed a molecular analysis of these genes in clinical isolates to determine the prevalence of known or novel mutations. Here, we describe the genetic analysis outcome. Furthermore, a biocomputational analysis of the *Mt*GidB L101F variant, the product of a novel mutation detected in *gidB* during molecular analysis, is also reported as a theoretical approach to study the apparent genotype-phenotype association.

## 1. Introduction

Worldwide, tuberculosis (TB) is among the top 10 causes of death and the leading disease caused by a single infectious agent, *Mycobacterium tuberculosis*, ranking above HIV/AIDS [1,2]. TB control has become a global challenge due to the continued emergence of multidrug-resistant (MDR) strains [3,4]. Precise identification of such strains demands bacterial confirmation and drug resistance assessment using culture methods, molecular analysis and DNA sequencing [5,6]. Moreover, patients with MDR-TB require intensive treatment for at least nine months (up to 20 months), supported by constant pharmacovigilance to reduce adverse events [7,8].

Streptomycin (Str) was the first antibiotic used for the therapeutic control of TB [9,10]. However, drug-associated side effects and the emergence of Str-resistant (Str^R^) strains prompted its removal from the group of first-line anti-TB drugs [11,12,13]. Despite this, WHO recommends using it in patients infected with a confirmed Str-susceptible strain [8,14,15]. Str is active against growing bacteria by inhibiting protein synthesis. It acts through irreversible binding to S12 protein and 16S rRNA, two molecular constituents of the small subunit of bacterial ribosomes [13,16].

Mutations in the *M. tuberculosis rpsL*, *rrs* and *gidB* genes, respectively, encoding the ribosomal protein S12, 16S rRNA and glucose-inhibited division protein B, have been associated with Str resistance [13,17,18,19,20,21]. The non-synonymous substitutions K43R and K88R in *rpsL* and nucleotide variations in the «530 loop» and «912 region» of 16S rRNA are the most frequent Str^R^-linked mutations. Remarkably, non-synonymous substitutions in *gidB* are usually associated with low-resistance levels [19,20,21,22,23,24,25].

The *gidB* gene product is an S-adenosylmethionine (SAM)-dependent 7-methylguanosine methyltransferase specific for 16S rRNA [26]. In particular, the GidB-catalyzed methylation occurs at nucleoside G527 of the *E. coli* 16S rRNA, which corresponds to G518 in the *M. tuberculosis* counterpart [27]. Thus, given that Str interacts with such a nucleoside, an impaired GidB function would affect the G518 methylation status of *M. tuberculosis* 16S rRNA, interfering with drug binding and, consequently, producing the observed Str^R^ phenotype [21,25,26,27,28].

Herein, we performed a molecular analysis of the *rpsL*, *rrs* and *gidB* genes in clinical isolates of *M. tuberculosis* that showed a noticeable Str^R^ phenotype to determine the current prevalence of known or novel mutations in our region. The combined outcome of DNA sequencing and gene comparisons allowed us to identify point mutations associated with such drug resistance. Furthermore, a biocomputational analysis was used as a theoretical approach to study the apparent genotype-phenotype association observed in two Str^R^ isolates containing the 301c>t mutation in *gidB*, which produces the non-synonymous substitution L101F in the gene product.

## 2. Results and Discussion

### 2.1. Molecular Analysis of the M. tuberculosis rpsL, rrs and gidB Genes

Patients suspected of having active TB disease who attended the GHT’s TB Clinic provided the sputum samples. From a collection period of one year, 11 clinical isolates met the inclusion criteria for this study: positive Ziehl-Nielsen (ZN) staining and streptomycin resistance (minimum inhibitory concentration, MIC > 0.8 µg/mL). Using standard molecular methods for *M. tuberculosis* gDNA extraction and PCR amplification, the expected gene fragments for molecular analysis: 628 bp (*rpsL*), 645 bp (*rrs*) and 719 bp (*gidB*), were obtained. Once purified, the amplicons were sequenced and analyzed with various bioinformatics tools: NCBI’s BLAST, SequentiX’s DNA Dragon, SnapGene^®^ Viewer and EBI’s Clustal Omega. Table 1 summarizes the data obtained from dsDNA sequencing and gene analysis.

As initial analysis of the molecular results, none of the Str^R^ isolates showed mutations in *rpsL*, i.e., fully identical to the reference gene sequence (Mycobrowser ID: Rv0682). Furthermore, most of them (10 out of 11) showed 100% identity within the 365–978 nucleotide segment of the reference *rrs* gene (Mycobrowser ID: MTB000019). The exception isolate (03R) showed the 491c>t mutation, which was also detected in Str-susceptible isolates, confirming a lack of genotype-phenotype association. Moreover, it represents an epidemiological biomarker assigned to the *M. tuberculosis* Latin American and Mediterranean sublineage 3 (LAM3) [28].

In contrast, the *gidB* analysis produced mixed results. Five isolates, 03R-07R, showed sequences 100% identical to the reference (Mycobrowser ID: Rv3919c). Another three: 02R, 09R and 11R, showed the 236t>c mutation (causing the L79S substitution). This variant is associated with a low-level Str^R^ phenotype when detected as a sole mutation. However, when concurring with mutations in other genes (e.g., *rrs* 517c>t or *rpsL* K43R), isolates exhibit significant resistance to Str [24,29,30,31]. Two others (R01 and R10) showed the 301t>c mutation (causing the L101F substitution). Knowledge about this gene variant and its contribution to the phenotype is limited. However, a common feature of Str^R^ isolates containing such a variation is the absence of mutations in their *rrs* and *rpsL* genes, suggesting a genuine association with the observed phenotype [29]. Lastly, isolate 08R showed two mutations, 37g>c and 47t>g, causing the G13R and L16R substitutions. While G13R seems to be a novel mutation, L16R is a natural polymorphism associated with the LAM lineage [20,24,29,31,32].

### 2.2. Significance of the Molecular Analysis

So far, the observed results allow us to separate the analyzed Str^R^ isolates into two groups (I and II, Table 1). Group I comprises of those lacking mutations in the commonly associated genes (i.e., *rpsL*, *rrs* and *gidB*) and those with mutations previously identified as polymorphisms (e.g., 491c>t in *rrs*). Interestingly, the identification of this group implies the existence of additional genes associated with the phenotype, as suggested by proteomic analyses [33,34,35]. On the other hand, group II includes those containing mutations in *gidB* and lacking known genetic variations in *rpsL* and *rrs*. Furthermore, while a subset of this group involves those carrying a mutation associated with a low level of resistance (i.e., L79S), another subset contains those revealing either G13R or L101F as a novel non-synonymous substitution.

Regarding the latter, G13R is within the N-terminal domain, and L101F is in the SAM-dependent methyltransferase (SAM-MTase) domain, within the SAM-interacting region [25,36,37]. As there is no prior evidence regarding the G13R genotype contribution to the Str^R^ phenotype, the effect of such a mutation on *Mt*GidB function will not be analyzed further. However, to gain additional knowledge on the molecular basis of Str resistance in *M. tuberculosis*, we performed a biocomputational analysis of the *Mt*GidB L101F variant to predict its functional consequences (given the conserved structure-function relationship among SAM-MTase domains).

### 2.3. Biocomputational Analysis of the M. tuberculosis GidB L101F Variant

#### 2.3.1. Structural and Functional Analysis

A primary structure-based biocomputational analysis of *Mt*GidB provided the initial information about the effect of the L101F substitution on protein function (Table 2). The combined results of three bioinformatics tools predicted a negative effect of such a mutation on *Mt*GidB function.

**Figure 1:**
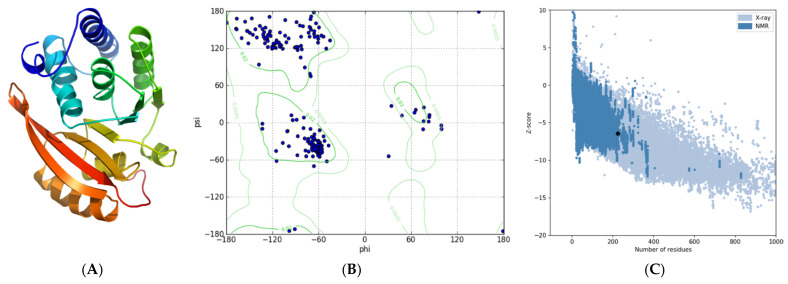
Predicted tertiary structure for *Mt*GidB. (**A**) Best 3D model (ribbon representation, rainbow-colored using default settings). (**B**) Ramachandran plot. (**C**) ProSA analysis (Z-score plot). A black dot denotes the estimated Z-Score.

Template-based modeling resulted in a reliable 3D structure to test whether L101F affects the functional conformation of *Mt*GidB, as suggested before. The predicted 3D model showed a high confidence score (Figure 1A): 1.17 (in the range −5 to 2, a higher value means high confidence). After refinement, the quality assessment result validated its structural accuracy: a global score of 0.7045 (values ≥ 0.4 are good scores). Furthermore, the Ramachandran plot showed that 89.1% of the non-Gly/Pro residues are in the most favored regions plus an additional 8.7% in allowed regions (Figure 1B). In addition, the estimated Z-score for overall quality, −6.44, is within the range of values typically found for proteins of similar size (Figure 1C).

Before further structure-function analysis, the predicted model served as a framework to identify the presumed SAM-interacting residues: G69, S70, G71, L74, E92, P93, L94, R97, G117, R118, A119, E120, R137 and A138. A tertiary structure-based bioinformatic analysis of the *Mt*GidB 3D model provided additional knowledge about the effect of the L101F substitution on protein stability. Overall, four biocomputational methods, along with a thermodynamic assumption: destabilizing (ΔΔG < −1.0 Kcal/mol), neutral (−1.0 ≤ ΔΔG ≤ 1.0 Kcal/mol) and stabilizing (ΔΔG > 1.0 Kcal/mol) [38], confirmed structural destabilization (Table 3).

#### 2.3.2. Polymorphic Site Interaction Analysis

**Figure 2:**
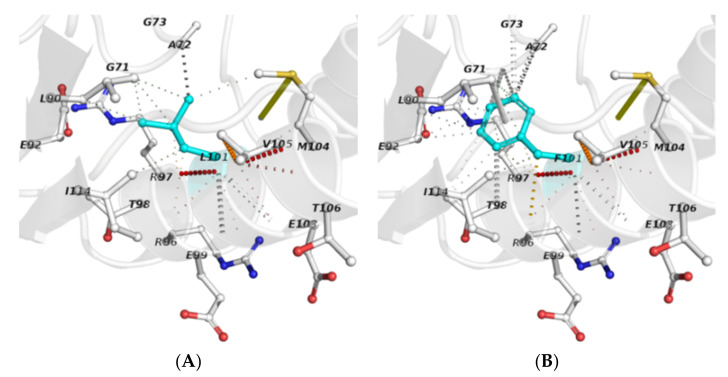
Interatomic contacts among residues that occur at the polymorphic site: (**A**) L101 (wild-type) and (**B**) F101 (mutant). Polymorphic residues (sticks) are depicted in cyan, while the others are displayed using the element-coloring settings. Non-covalent interactions are presented in default colors, as stated in the Arpeggio server.

The analysis of interatomic contacts at the polymorphic site provided further insights into the local interactions and changes derived from residue substitution (Figure 2). In this regard, a comparative examination of the respective networks of non-covalent bonds revealed that the mutant residue (F101) establishes new interactions and loses others, in contrast to the wild-type (L101). A supplementary analysis of interatomic contacts of structural units confirmed this observation (Table 4). Furthermore, as L101 is involved in a residue contact network that includes SAM-interacting residues (i.e., G71, E92 and R97), it seems reasonable to suggest that the L101F substitution affects the *Mt*GidB function by altering the ligand-binding site of the SAM-MTase domain.

#### 2.3.3. Protein Dynamics Analysis

**Figure 3:**
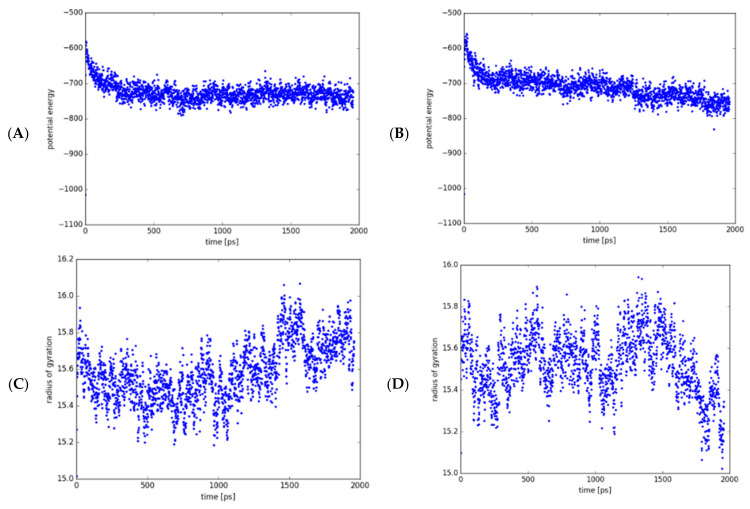
MD analysis of the wild-type (WT, L101) and mutant (MU, F101) systems (*Mt*GidB) in a 2000-ps simulation. Plots of potential energy (Kcal/mol) for WT (**A**) and MU (**B**). Plots of the radius of gyration (Å) for WT (**C**) and MU (**D**).

Molecular dynamics (MD) simulations are commonly applied to study protein mobility and flexibility [39,40]. Using coarse-grained (CG) models as reduced representations of protein residues, this theoretical approach provided additional knowledge about the conformational structure of *Mt*GidB and its changes due to the L101F substitution in a 2000 ps time frame. Interestingly, both systems (wild-type and mutant) depicted a short phase of continuous decrease in the UNRES (united residue) potential energy followed by an apparent steady-state, which remained until the simulation end (Figure 3A,B). However, the radius of gyration plots showed that the mutant system exhibits a higher degree of structural mobility than the wild-type system (Figure 3C,D), supporting the hypothesis that implies changes in local flexibility are the consequence of the L101F mutation on the *Mt*GidB conformation.

**Figure 4:**
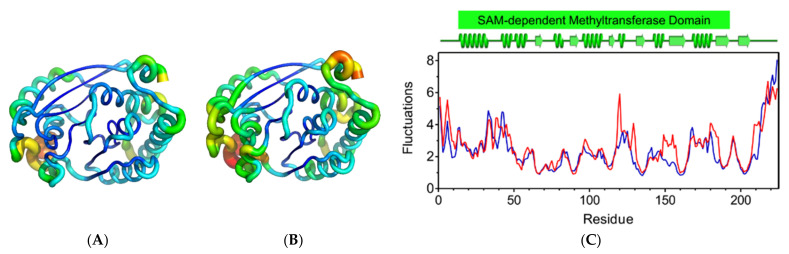
Analysis of atomic fluctuations. Cartoon putty representations of *Mt*GidB structures: (**A**) wild-type (WT, L101) and (**B**) mutant (MU, F101). Blue represents the lowest value for B-factor and red the highest. The size of the tube reflects the value of the B-factor (i.e., the larger the B-factor, the thicker the tube). (**C**) A plot of the residue-based fluctuations (Å) estimated for WT (blue) and MU (red). The predicted secondary structure (α-helices and β-strands) for *Mt*GidB and the stretch corresponding to its SAM-MTase domain are colored green (PDB representation, top panel).

Supplementary analysis of atomic fluctuations completed the knowledge on residue-based flexibility. Even though both retained the secondary structure, the mutant system showed increased overall flexibility than the wild-type system (Figure 4A,B), with significant deviations in residues and regions of the SAM-MTase domain (Figure 4C). This structural behavior suggests that the L101F substitution indirectly affects the flexibility of other residues and, thus, the global *Mt*GidB structural stability.

### 2.4. Significance of the Biocomputational Analysis

Overall, the MD results suggest that the L101F mutation affects the flexibility and stability of the *Mt*GidB structure, probably due to local and global intramolecular perturbations. Furthermore, as the L101 residue is involved in a contact network that includes SAM-interacting residues (i.e., G71, E92 and R97), it seems reasonable to suggest that the Leu>Phe substitution at position 101 affects the *Mt*GidB function by altering the ligand-binding site of the SAM-MTase domain. However, an experimental approach is required to test the latter hypothesis and accurately establish the genotype-phenotype association observed in the clinical isolates of *M. tuberculosis*.

## 3. Materials and Methods

### 3.1. Sample Collection and Mycobacteriological Analysis

Sputum samples from patients suspected of having active TB were collected at the TB Clinic of the General Hospital of Tijuana (GHT) by qualified personnel. All samples were digested-decontaminated using a BBL MycoPrep™ System (Becton Dickinson). The mycobacteriological analyses and drug-susceptibility assays were performed at the TB Diagnostic Unit (GHT), using standard protocols. Out of 157 independent samples from a one-year collection period, 11 tested positive for two inclusion criteria: acid-fast bacilli by Ziehl-Nielsen (ZN) staining and Str-resistant (MIC > 0.8 µg/mL) by MGIT analysis (BACTEC 960 System, Becton Dickinson). In this case, 15 ZN-positive Str-sensitive samples, selected at random, were used as controls. Sample handling and subsequent procedures were according to standard protocols approved by the GHT’s Ethics Committee.

### 3.2. Molecular Methods

#### 3.2.1. Mycobacterial DNA Extraction

Genomic DNA was isolated using the DNAzol reagent (Becton Dickinson) and the protocol recommended by the manufacturer. Briefly, 0.2 mL of MycoPrep’s sediment and 0.5 mL of 1X Dulbecco’s PBS solution were mixed, and bacterial lysis was completed by heating at 80 °C (10 min). After cooling for 1 min on an ice bath, 1 mL of DNAzol reagent was added, mixed thoroughly, and centrifuged at 10,000 rpm for 10 min (to remove cell debris). The supernatant was mixed with 0.5 mL of cold ethanol and then centrifuged for 10 min at 14,500 rpm. The precipitated DNA was air-dried for 10 min and then dissolved in 30 µL of 8 mL NaOH.

#### 3.2.2. PCR Amplification of Gene Fragments

Typical PCR reactions (20 μL) contained 10 picomoles of each primer (i.e., Fw/Rv) and 1 µL of template DNA in 1X *Taq* Master Mix (New England Biolabs). Table 5 lists the synthetic oligonucleotides used as primers for PCR amplification of the *M. tuberculosis rpsL*, *rrs*, *gid*B gene fragments. Reactions were completed in a C1000 Touch™ Thermal Cycler (Biorad) using the following settings: an initial denaturation step (2 min at 94 °C), 45 cycles of exponential amplification (20 s at 94 °C, 20 s at 55 °C, 20 s at 72 °C) and a final extension step (7 min at 72 °C). A reaction lacking template DNA was used as a negative control, while another containing *M. tuberculosis* H37Rv genomic DNA (1 ng) was positive (and reference DNA for comparative purposes).

#### 3.2.3. Analysis and Purification of Amplicons

The amplification products were analyzed by 2.0% agarose gel electrophoresis, using EtBr as a fluorescent dye (0.5 µg/mL, final), and visualized/documented with a GelDoc™ EZ Imager (Biorad). The 100-bp DNA Ladder and λ-DNA/HindIII Digest (New England Biolabs) were the DNA markers used to assess molecular weights. The amplicons: 628 bp for *rpsL*, 645 bp for *rrs* and 719 bp for *gidB*, were purified using a QIAquick PCR Purification Kit, as recommended by the manufacturer (Qiagen).

#### 3.2.4. DNA Sequencing and Data Analysis

All Amplicons were sequenced by the Sanger method using gene-specific primers (Table 5) and a SeqStudio™ Genetic Analyzer (ThermoFisher Scientific). The NCBI BLAST engine (https://blast.ncbi.nlm.nih.gov/Blast.cgi, accessed on 1 September 2020) [41] was the computational tool used to perform DNA sequence comparisons against *M. tuberculosis* H37Rv (as a reference). The double-stranded DNA sequences were analyzed using two different biocomputational packages, the SequentiX’s DNA Dragon—Sequence Contig Assembler (https://www.dna-dragon.com/, accessed on 15 September 2020) and SnapGene^®^ Viewer (https://www.snapgene.com/snapgene-viewer/, accessed on 15 September 2020). The multi-sequence alignments were generated by Clustal Omega [42,43], using the EMBL-EBI server (https://www.ebi.ac.uk/Tools/msa/clustalo/, accessed on 15 September 2020).

### 3.3. Biocomputational Methods

#### 3.3.1. Sequence-Based Function Predictions

Three bioinformatic predictors determined the effect of L101F substitution on the function of *Mt*GidB: PolyPhen-2, PROVEAN and SIFT, with default settings. PolyPhen-2 (http://genetics.bwh.harvard.edu/pph2/, accessed on 1 October 2020) is an algorithm that combines sequence and structure-based attributes and uses a naive Bayesian classifier to identify missense mutations with an impact on the phenotype. Output levels of probably (0.85–1.0) and possibly (0.15–0.84) damaging are significant [44,45,46]. PROVEAN (http://provean.jcvi.org/, accessed on 1 October 2020) is an alignment-based method that estimates the influence of amino acid substitutions on protein function. The final score designates the mutation as deleterious or neutral, according to a predefined threshold. Protein variants with a score equal to or less than −2.5 are deleterious [47,48]. SIFT (https://sift.bii.a-star.edu.sg/, accessed on 1 October 2020) is a sequence homology-based tool that classifies amino acid substitutions as tolerant (neutral) or intolerant (deleterious) mutations. Protein variants with a normalized probability value equal to or less than 0.05 are deleterious [49].

#### 3.3.2. Template-Based Protein Modeling

The three-dimensional (3D) structure of *Mt*GidB was generated by template-based modeling using the I-TASSER server (https://zhanglab.ccmb.med.umich.edu/I-TASSER/, accessed on 1 November 2020), a unified platform that uses a hierarchical approach for automated 3D structure prediction [50,51,52]. C-score was used to measure the modeling confidence [53,54]. The top-ranked 3D structure was improved using ReFOLD (http://www.reading.ac.uk/bioinf/ReFOLD/, accessed on 10 November 2020), a computational tool for model refinement guided by accurate quality estimates [55,56], and the structural precision was estimated using ModFold (https://www.reading.ac.uk/bioinf/ModFOLD/, accessed on 20 November 2020), a server for global and local quality assessment [57]. The 3D model was further analyzed using Procheck’s Ramachandran plot [58] and ProSA’s Z-score plot [59,60]. PyMol (Schrödinger, LLC.) and UCSF Chimera [61] were the molecular graphics systems used to visualize protein structures.

#### 3.3.3. Structure-Based Stability Predictions

Five biocomputational methods predicted the effect of L101F substitution on the stability of *Mt*GidB: DeepDDG, DUET, mCSM and SDM, with default settings. DeepDDG (http://protein.org.cn/ddg.html, accessed on 5 December 2020) employs a well-trained, neural network-based method to predict changes in protein stability due to point mutations [62]. DUET (http://biosig.unimelb.edu.au/duet, accessed on 5 December 2020) predicts the effects of missense mutations on protein stability by combining two complementary approaches in a consensus prediction [63,64]. mCSM (http://biosig.unimelb.edu.au/mcsm, accessed on 5 December 2020) uses graph-based signatures to predict the impact of missense mutations on protein stability, encoding distance patterns between atoms [65]. SDM (http://marid.bioc.cam.ac.uk/sdm2, accessed on 5 December 2020) applies conformationally constrained environment-specific substitution tables to predict the effect of a missense mutation and calculate the change in protein stability [66,67].

#### 3.3.4. Examination of the Interatomic Contacts

The interatomic contacts at the polymorphic site were estimated using Arpeggio (http://biosig.unimelb.edu.au/arpeggioweb/, accessed on 15 December 2020), a web service for calculating the interatomic interactions in protein structures [68]. The *Mt*GidB model was the wild-type structure, and its L101F variant the mutant. The local network of non-covalent interactions was analyzed using the PyMol system. The specific contacts were detected using the LPC/CSU software (http://oca.weizmann.ac.il/oca-bin/lpccsu, accessed on 15 December 2020) by automatic analysis of interatomic contacts of structural units (CSU) [69].

#### 3.3.5. Coarse-Grained Molecular Dynamics Simulations

Coarse-grained (CG) models for (MD) simulations are an effective biocomputational approach for adequate sampling of the conformational space while maintaining physical rigor [70]. The CG-MD simulations were performed online by the UNRES web server (https://unres.pl/, accessed on 10 January 2021), using the *Mt*GidB 3D model as a wild-type structure and its L101F variant as the mutant structure with default settings for standard protein dynamics. The CG united residue (i.e., UNRES) model is a highly-reduced physics-based representation of proteins, in which only two interaction sites per residue (united side chains and united peptide groups) are present [71,72,73]. The automatic output data, such as plots of potential energy and radius of gyration, were downloaded and analyzed as generated by the server. The fluctuations results were analyzed using the Pymol system.

## Figures and Tables

**Table 1:** Overall results of the molecular analysis of *rpsL*, *rrs* and *gidB* genes from 11 clinical isolates of *M. tuberculosis* showing resistance to streptomycin.

Isolate ID	*rpsL*	*rrs*	*gidB*	Group ^§^
01R	ND	ND	301c>t (L101F)	II
02R	ND	ND	236t>c (L79S)	II
03R	ND	491c>t *	ND	I
04R	ND	ND	ND	I
05R	ND	ND	ND	I
06R	ND	ND	ND	I
07R	ND	ND	ND	I
08R	ND	ND	37g>c (G13R); 47t>g (L16R)	II
09R	ND	ND	236t>c (L79S)	II
10R	ND	ND	301c>t (L101F)	II
11R	ND	ND	236t>c (L79S)	II

ND: no mutation detected (i.e., 100% identical to the reference DNA sequence). * Mutation shared with streptomycin-sensitive clinical isolates. ^§^ Grouped by in-house criteria.

**Table 2:** Effect of L101F substitution on *Mt*GidB function.

Method	Score	Cutoff	Prediction
PolyPhen-2	1.00	≥0.85	Probably Damaging
PROVEAN	−3.98	≤−2.5	Deleterious
SIFT	0.00	≤0.05	Affect Function

**Table 3:** Effect of L101F substitution on *Mt*GidB stability.

Method	ΔΔG (Kcal/mol)	Prediction ^§^	Assumption
DeepDDG	−1.83	Destabilizing	Destabilizing
DUET	−1.75	Destabilizing	Destabilizing
mCSM	−1.48	Destabilizing	Destabilizing
SDM	−1.39	Destabilizing	Destabilizing

^§^ As returned by the computational algorithm.

**Table 4:** Residues in contact with L101 (wild-type) or F101 (mutant) in the *Mt*GidB 3D structure. Classification of non-covalent interactions as detected by the LPC/CSU software program.

Residue		Specific Contacts with L101						Specific Contacts with F101					
		D (Å)	S (Å^2^)	HB	Arm	Pho	DC	D (Å)	S (Å^2^)	HB	Arm	Pho	DC
71	Gly	4.8	4.5	−	−	−	+	3.4	33.4	−	−	−	−
72	Ala	3.5	32.3	−	−	+	+	3.2	13.9	−	−	+	−
73	Gly	3.8	7.9	−	−	−	+	3.2	26.7	−	−	−	−
90	Leu	3.7	31.4	−	−	+	−	4.1	23.1	−	−	+	+
92	Glu	4.9	11	−	−	+	−	3.5	28.9	−	−	−	−
97	Arg	2.9	19.4	−	−	+	+	2.9	12.6	−	−	−	+
98	Thr	3.5	20.6	−	−	−	+	3.1	31	−	−	−	−
100	Phe	1.3	91	+	−	+	+	1.4	82.5	+	+	−	+
102	Arg	1.4	52.5	+	−	−	+	1.3	52.9	−	−	−	+
104	Met	3.1	12.8	−	−	+	+	3.3	8.2	−	−	+	−
105	Val	2.8	40.7	−	−	+	+	2.8	40.6	−	−	+	−
114	Ile	4.3	11.7	−	−	+	+	3.9	20	−	−	+	−

D, the nearest distance between atoms of two residues; S, contact surface area between two residues; HB, hydrophilic-hydrophilic contact (hydrogen bond); Arm, aromatic-aromatic contact; Pho, hydrophobic-hydrophobic contact; DC, hydrophobic-hydrophilic contact (destabilizing contact); +/−, presence/absence of a specific contact.

**Table 5:** Gene-specific primers for PCR amplification or DNA sequencing.

Gene	Primer ^§^	Sequence (5′→3′)	Application
*rpsL*	MTRPSLF1	gatgcctcggatgagacgaatc	PCR amplification
	MTRPSLR1	taaacaatgcgctcggccag	PCR amplification
	MTRPSLF2	cgagtttgaggcaagctatg	DNA sequencing
	MTRPSLR2	cccttcaacagaaccttgttcac	DNA sequencing
*rrs*	MTRRSF1	agtggggaatattgcacaatgg	PCR amplification
	MTRRSR1	gtcctgtgcatgtcaaacccag	PCR amplification
	MTRRSF2	attgcacaatgggcgcaagc	DNA sequencing
	MTRRSR2	ggtaaggttcttcgcgttgc	DNA sequencing
*gidB*	MTGIDBF1	cacagacctcacgagccgg	PCR amplification
	MTGIDBR1	gccccacggagcactcac	PCR amplification
	MTGIDBF2	ccggcggagtgcgtaatg	DNA sequencing
	MTGIDBR2	gcactcacgccgtccctc	DNA sequencing

^§^ Obtained from Eurofins Genomics LLC (Louisville, KY).

## Data Availability

All data presented in this study are available on request from the corresponding author, without undue reservation, to any qualified researcher.
